# Saving Saba Bank: Policy Implications of Biodiversity Studies

**DOI:** 10.1371/journal.pone.0010769

**Published:** 2010-05-21

**Authors:** Paul C. Hoetjes, Kent E. Carpenter

**Affiliations:** 1 Department of Environment and Nature, Directorate of Public Health, Willemstad, Curaçao, Netherlands Antilles; 2 International Union for Conservation of Nature Species Programme/Species Survival Commission and Conservation International Global Marine Species Assessment, Biological Sciences, Old Dominion University, Norfolk, Virginia, United States of America; University of Hull, United Kingdom

## Abstract

Saba Bank has always been an area of special importance to the neighboring island of Saba in the Netherlands Antilles. Sabans traditionally fished on the Bank as far back as 1907, but increasing foreign fishing pressures on the Bank in the 1970s and 1980s forced many Saban fishermen out. Concerns were compounded by the suspicion that shipping was also damaging the benthic habitat of the bank. Fishery legislation, enacted in 1996, brought an end to unlicensed fishing and established Coast Guard enforcement on the Bank, but also led to protests from neighboring countries that previously fished on the Bank.

Research was necessary to support the need for protection. Review of available research of Saba Bank and rapid biological assessments and fisheries surveys since 1996 emphasized the richness of Saba Bank's biodiversity and the need for protection of fisheries stocks. The national nature policy plan recognized this and encouraged further research to base conservation measures on.

Recent biological surveys of corals, fishes, and algae presented in this collection of articles emphasized habitat heterogeneity and the relative richness of the marine flora and fauna. These assessments formed the basis for a management plan to protect Saba Bank's biodiversity and a draft proposal to the International Maritime Organization (IMO) seeking Particularly Sensitive Sea Area (PSSA) status for the Bank. The intention of the PSSA proposal is to protect the benthic habitat on Saba Bank from anchor damage. This paper serves to provide the context for the results of the recent biodiversity surveys of Saba Bank. It is hoped that this collection will serve as a knowledge baseline and engender further research in the area.

## Introduction

**Figure 1:**
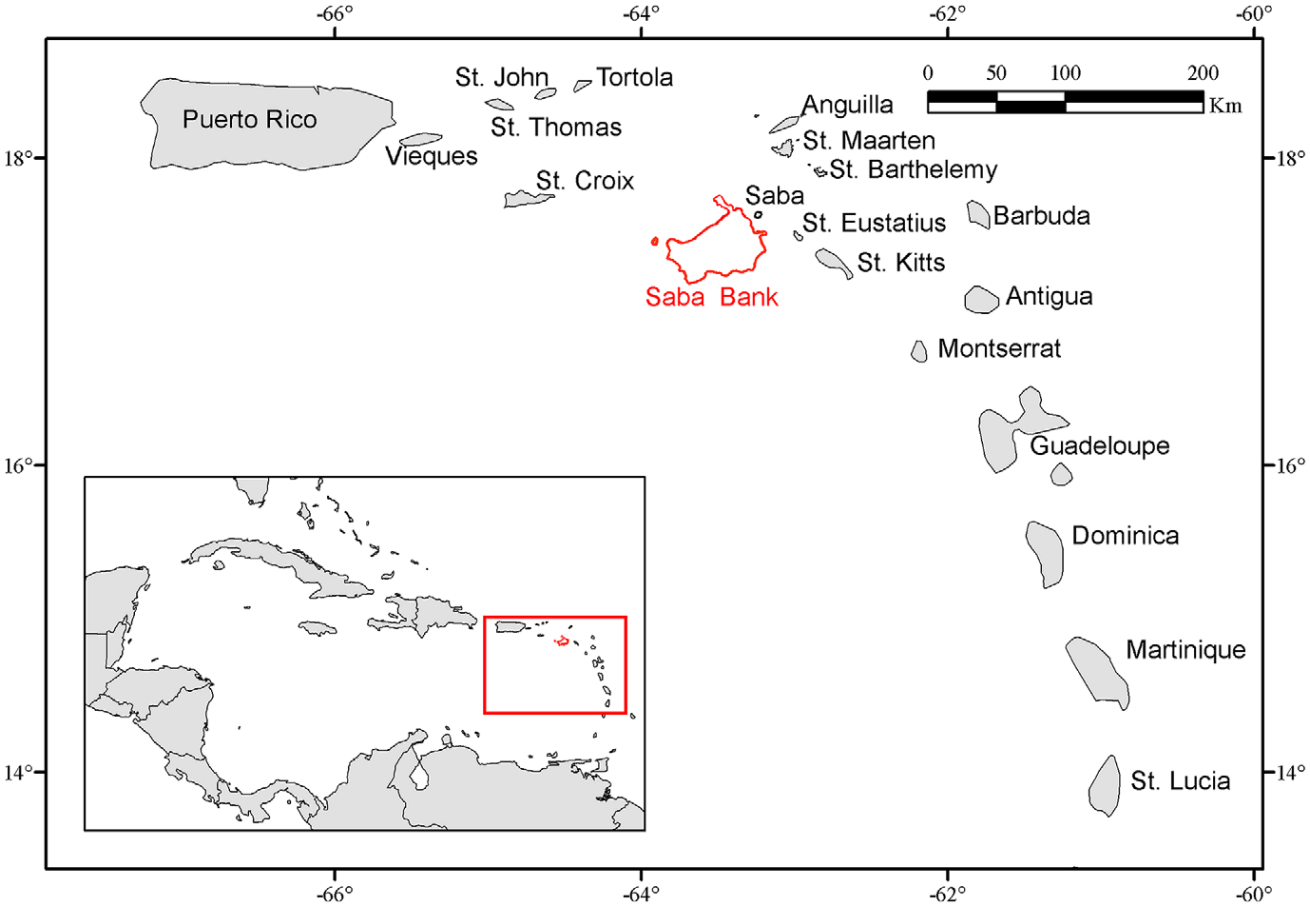
Position of Saba Bank within the Caribbean region. Saba Bank is part of the inner arc of the Lesser Antilles.

Saba Bank is a large shallow area rising steeply out of deep oceanic waters in the northeastern Caribbean, located about 6 km south of the island of Saba (Fig. 1). The shallow top of the bank is remarkably flat and has an area of roughly 2000 km^2^ with a depth of less than 50 m. It is roughly rectangular in outline, measuring approximately 40 by 60 km with its long axis oriented southwest to northeast. Average depth is about 25 m with a shallower ridge along the eastern and southeastern edges of about 15 to 18 m depth while sloping gradually deeper towards the west.

**Figure 2:**
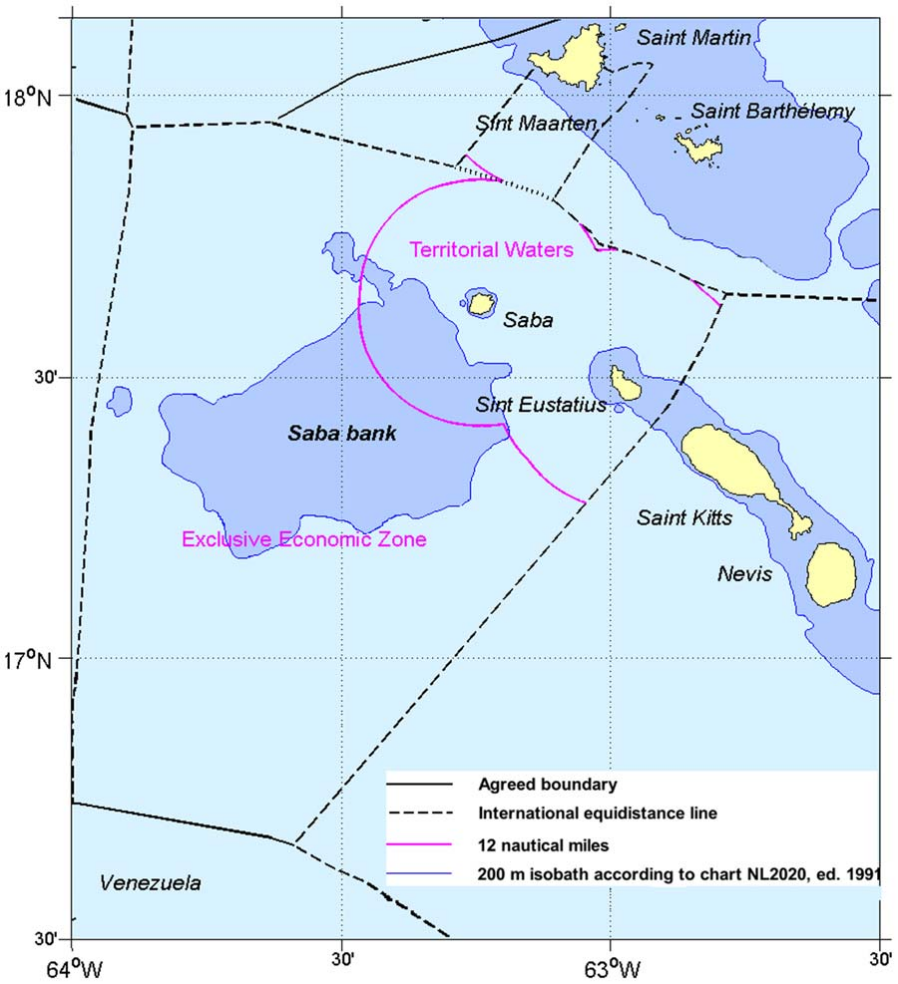
Saba Bank and surrounding islands, with territorial waters and EEZ boundaries. The Netherlands Antilles is currently in the process of declaring its Exclusive Economic Zone. The boundaries of this EEZ coincide with those of the current Exclusive Fisheries Zone (EFZ) and consist in most cases of accepted international equidistance lines between countries; only the boundary with Venezuela has been agreed and specified in a bilateral treaty between the Netherlands and Venezuela. About 20% of Saba Bank falls within the “twelve-miles zone” or territorial waters of the island of Saba, the rest falls completely within the EEZ boundaries and thus under direct federal jurisdiction of the Netherlands Antilles.

For generations Saba Bank has been fished by the Sabans. This fishery was first documented early in the twentieth century by Boeke [1] who also mentioned the existence of extensive coral growth. The Bank intrigued a number of scientists since the early twentieth century who studied and debated its geology [2], [3], [4], [5], [6], but otherwise little attention was given to Saba Bank until the nineteen seventies when many Caribbean nations declared Exclusive Economic Zones (EEZ) and started to control their fisheries. As a consequence Saba Bank became a refuge for foreign fishing vessels that had been excluded from other islands [7]. As the Netherlands Antilles did not declare an Exclusive Fisheries Zone (EFZ) until 1993 it did not have jurisdiction over fisheries on Saba Bank except within the territorial waters of Saba extending 12 nautical miles out from the island that included about 20% of the Saba Bank. After the declaration of an EFZ, which included the whole of Saba Bank, the management of the Bank, in particular of its fishery, became a responsibility of the Netherlands Antilles government. **Figure 2** shows the position of Saba Bank relative to the surrounding islands with the boundaries of the territorial waters of the Dutch islands and of the EEZ.

Indirect evidence suggested that the Bank was being overfished. In 1994 for instance, just after the EFZ was declared, export permits were requested under CITES (Convention on the International Trade of Endangered Species) regulations for as many as ten container loads of conch (*Strombus gigas*) meat, a total of about 200,000 kg (200 metric tons) amounting to close to a million individuals that could only have come from the Saba Bank, since there are no other significant stocks in the Netherlands Antilles. By comparison, the mean annual catch reported between 1993 and 2001 by Belize—where conch is considered to be overexploited and with a coastal area at least five times greater than Saba Bank—was also about 200 metric tons [8]. The fishermen on Saba were complaining bitterly about the large foreign fishing vessels that were emptying the fish stocks of the Bank, sometimes threatening the small fishing boats from Saba, and in fact many local Saban fishermen stopped fishing because of this [7].

Enforcement of fisheries regulations improved with the active patrolling of Saba Bank by the Coast Guard of the Netherlands Antilles and Aruba, which effectively ended illegal foreign fishing. This in turn led to complaints from some of the countries of origin of fishing vessels that could no longer fish on Saba Bank, and requests to provide fishing permits for these vessels. This was denied on the grounds of insufficient information being available about the fish stocks, a valid reason but only as long as an effort is being made to collect such data and until such time as conclusions can be drawn from those data. In the case of CITES permits for export of Queen Conch, CITES regulations prohibit such export without a clear finding of “non-detriment” to the species; data on this were completely lacking.

There was a clear need for data on the biodiversity of Saba Bank in order to formulate a policy to adequately protect the biodiversity of Saba Bank including its fish stocks, and this is what the Netherland Antilles Department of Environment and Nature (MINA) set out to do.

## Results and Discussion

### Research and policy on Saba Bank

The first step to find out more about Saba Bank was a review of the existing knowledge and a quick field survey of the Bank, commissioned by MINA. This was completed in 1996. While confirming that little was known about Saba Bank, this study by Meesters *et al.* concluded that it was an area of great interest, both geologically and biologically [9]. Meesters reports that, although different views exist, the most recent conclusion in 1977 by Van der Land [6] is that Saba Bank is an actively growing, though submerged atoll, and as such it is the largest in the Caribbean and the third largest in the world. Meesters also concludes that it is a regionally unique ecosystem, relatively pristine and remote from human influences, with high biological diversity and productivity, potentially an important source of fish and invertebrate larvae to the islands of Saba, Sint Maarten, the islands of the Greater Antilles and the Virgin islands. At the same time Meesters notes that there is a threat of overfishing and damage from anchoring by large tankers [9].

Meesters recommended further study of Saba Bank, improved legislative instruments, including international instruments, and enforcement to control current and future activities as well as capacity and awareness building [9]. This formed the basis for the Netherlands Antilles government's policy for Saba Bank. Lack of resources and capacity delayed implementation of this policy, but a first in-depth fisheries catch assessment was concluded in 2000 that provided solid data about the state of the fisheries [7]. This study concluded that no new fishing permits should be issued until a long term fishery monitoring program was in place, while emphasizing the need for effective enforcement of existing regulations [7]. This had immediate effects on Saba Bank policy, with the island government of Saba declaring a moratorium on fishing permits and the start of a capacity building effort to strengthen the Coast Guard's enforcement of existing regulations. In addition, the Department of Environment produced a video about Saba Bank, for the first time providing people with images of the Bank's unique underwater life, showing its importance to fisheries and emphasizing the need for protection.

Another aim of the policy with regard to Saba Bank was to develop international instruments to allow better control of activities on the Bank. The Netherlands Antilles intended to change the Exclusive Fisheries Zone (EFZ) declared by the Netherlands Antilles into an Exclusive Economic zone (EEZ), providing not only jurisdiction over fishery resources, but over all natural resources, including the biodiversity resources of Saba Bank. Another international instrument, in particular with respect to international shipping, was to request a special status for Saba Bank from the International Maritime Organization (IMO). A Particularly Sensitive Sea Area (PSSA) is an area that needs special protection through action by IMO because of its significance for recognized ecological, socio-economic, or scientific reasons and because it may be vulnerable to damage by international shipping activities [10]. Such a status would mark Saba Bank on nautical maps as a sensitive area, allow routing of tanker traffic around (parts of) Saba Bank, and anchoring prohibitions to reduce the serious impacts on bottom flora and fauna and on the fisheries. However, in order to successfully argue for PSSA status of Saba Bank further study of the Bank's biodiversity was needed in order to demonstrate that the area satisfies all the criteria adopted by the IMO.

The assistance of the Dutch Ministry of Transport and Water proved to be a key factor in this. They not only provided support for the drafting of new legislation that would allow declaration of an EEZ (as well as implementing various international environmental regulations), they also supported the White Water to Blue Water (WW2BW) initiative, an international alliance of governments, international organizations, financial institutions, non-governmental organizations (NGOs), universities, and corporations that aims to stimulate partnerships for integrated watershed and marine ecosystem-based management. This initiative was created at the World Summit on Sustainable Development (the Johannesburg Summit) in 2002, and formally launched at a Wider Caribbean “Partnership Conference” held in Miami in March 2004. The intentions of the Netherlands Antilles for Saba Bank had previously been discussed with Conservation International, which had just identified the Caribbean as a global biodiversity hotspot. The 2004 WW2BW conference specifically addressed the possibilities and requirements for PSSA status of marine protected areas and formed the perfect forum to announce a WW2BW partnership of the Netherlands Antilles, Conservation International, and the National Oceanographic and Atmospheric Agency (NOAA) of the USA for the purpose of the protection of Saba Bank through PSSA status.

**Figure 3:**
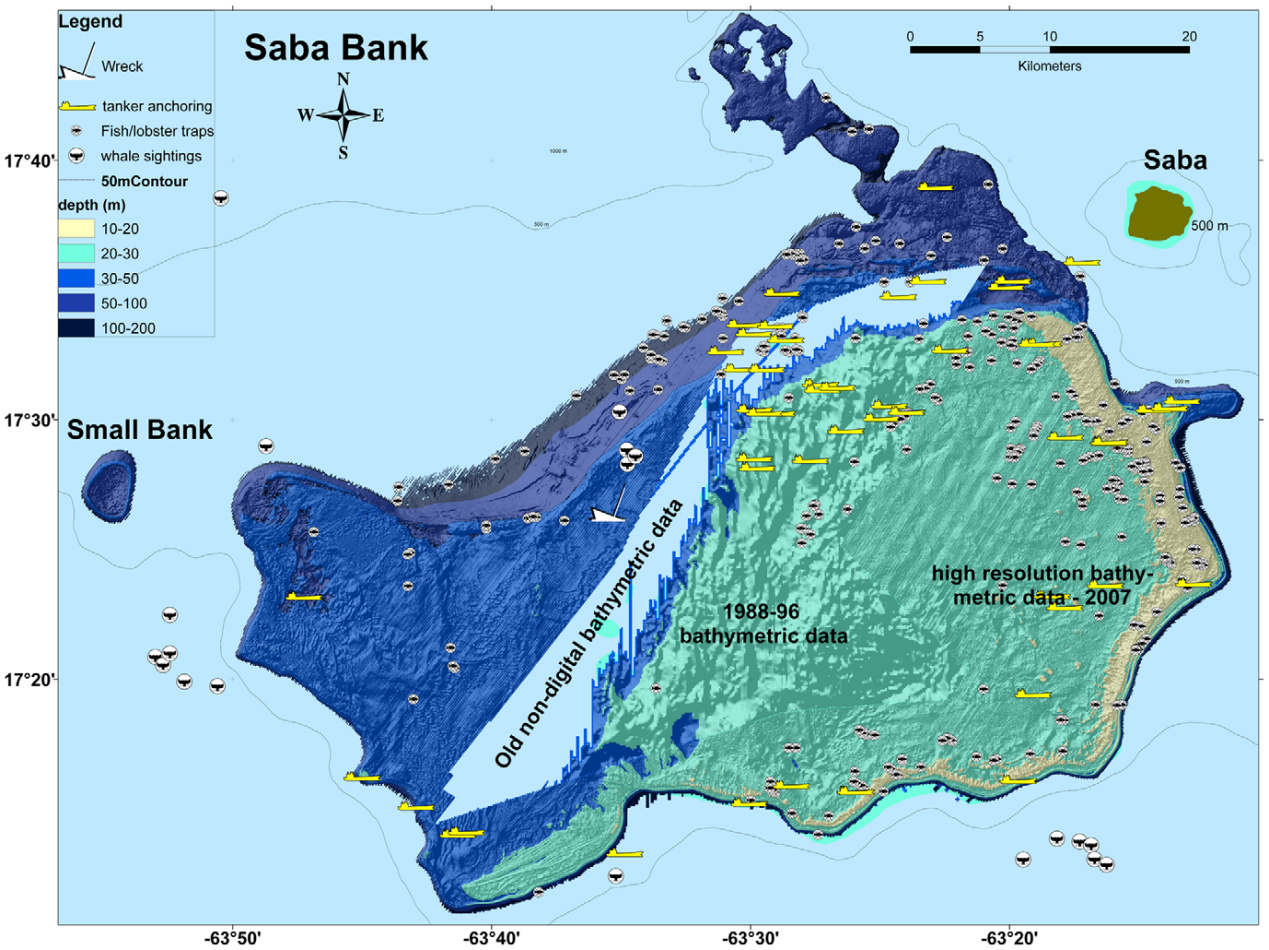
Saba Bank bathymetric map and human use. Various conflicting uses are made of Saba Bank. Fish and lobster traps are set over most parts of Saba Bank, while tankers routinely anchor in the same areas, not only damaging the bottom but also running over the trap markers and causing many traps to be lost. Anchoring also happens in the coral reef areas on the eastern edges. The map also shows whale sightings recorded by the vessel Song of the Whale during a regional survey in 2006, more research is needed on the cetaceans of the Saba Bank.

With the help of Conservation International, a Rapid Assessment Program (RAP) of Saba Bank was organized in 2006. Conservation International's Rapid Assessment Program was created in 1990 to quickly provide the biological information necessary to catalyze conservation action and improve biodiversity protection. The purpose of the program is to produce appropriate and realistic conservation recommendations in a time frame suited to managers and decision-makers [11] (http://science.conservation.org/portal/server.pt?open=512&objID=428&mode=2&in_hi_userid=124186&cached=true). It was this survey of Saba Bank's biodiversity that more than anything demonstrated the richness of its biodiversity. Not only were many species of fishes, corals and sponges found that had not been reported before, but Saba Bank was found to have a uniquely diverse marine macro-algal flora; including many new species of marine macro-algae never described before. Reports of the findings of this RAP expedition are published alongside this introduction in this special volume of PLoS One. The volume, dedicated to Saba Bank, includes characterizations of Saba Bank's extraordinary macroalgae communities, as well as assessments of the sponges, hard corals, and fishes, [12]–[15]. The expedition brought worldwide publicity for Saba Bank and helped to earmark Dutch development funding for further study of the Bank. Serendipitously, immediately after the RAP expedition the Dutch Navy carried out a hydrographic survey of Saba Bank as part of the regular updating of its nautical charts every ten years. Again with help of Conservation International, a high-resolution bathymetric GIS map of Saba Bank was produced in 2007 using the Navy's sonar data within a Geographic Information System (GIS) framework. This map formed the basis for further study of Saba Bank, including a detailed characterization of some of the bottom habitats and associated fish assemblages of the Bank, also presented here in this Saba Bank volume [16]. At the same time a second fisheries assessment could be undertaken to follow up on the previous study that by this time was 7 years old. Another expedition was organized, this time to assess the crustacean and gorgonian fauna of Saba Bank. The gorgonian survey, also in this Saba Bank volume, discovered two undescribed octocoral species and was able to distinguish two different shallow water gorgonian habitats [17]. In addition to these studies, the damage caused by anchoring tankers was documented in a few cases, while a vessel monitoring system on Saba provided the means to document the number of tankers anchoring on the Bank. **Figure 3** shows a bathymetric map of Saba Bank and various uses and features including fish/lobster trap positions, and anchoring tanker positions.

A management plan for Saba Bank was drafted with a more adequate body of research describing Saba Bank's biodiversity, more data on the use being made of the Bank, and a better idea of the habitats present on the Bank [18]. A PSSA status proposal was also drafted and is currently being finalized. New legislation to regulate international shipping in the waters of the Netherlands Antilles, needed in order to submit a PSSA proposal to the IMO, was passed. This legislation also makes it possible to declare an EEZ (another requirement for the PSSA proposal). The process is currently underway, and is expected to be finalized in 2010. The new legislation also prohibits anchoring by large ships in the territorial waters of Saba (20% of Saba Bank), but in 2010 the anchoring prohibition will be extended to the whole of the Bank. Once the means are found to implement the management plan, everything will finally be in place to protect the biodiversity of Saba Bank. Since an adaptive management framework based on an ecosystem approach was chosen for the management of Saba Bank, further study and monitoring of the Bank's biodiversity and use will be required to guide the Bank's management in a feedback loop.

Based on this example of Saba Bank the authors believe that interaction of biodiversity research and policy development is essential to developing effective management of biodiversity and public support.

The recent surveys of Saba Bank were part of a coherent effort to direct and to support conservation efforts, and to learn more about the area and therefore a dedicated peer-reviewed volume was sought to collect and disseminate these results. It was believed that such a collection of results could support the need for conservation better than scattered publications not only to illustrate the range and richness across taxa, but also to provide a consolidated baseline of scientific information about the biodiversity of Saba Bank. The open-access journal format lends further support to biodiversity conservation, because fishermen and managers have equal opportunity to review these results. Hopefully this volume will serve as a starting point and stimulus for further research into the various habitats of the Bank, about which far too little is as yet known.

To date, only a very small part of the huge area has been adequately sampled. A variety of bottom classes have been identified and mapped from side scan sonar data but further and wider surveys are needed to determine whether substrate classes are a suitable proxy for different biological habitats on Saba Bank. A project combining satellite imagery interpretation, side scan sonar bottom classes and groundtruthing is planned for 2010 to address this gap in our knowledge. Another priority is a study of marine mammals on Saba Bank to determine their presence and use of the bank. Anecdotal evidence suggests that humpback whales may use the Bank for calving, and sperm whales may find prey around the steep edges of the Saba Bank platform. Other areas of research that would contribute to more effective management include further studies of conch (*Strombus gigas*) and lobster (*Panulirus argus*) populations, and sea turtles' use of the Bank. Socio-economic studies would also be welcome. It is the hope of the authors that this Saba Bank volume will help to generate interest in further work and to attract more scientific publications that can be added on to this initial collection.
